# Molecular identification of *Bambusa changningensis* is the natural bamboo hybrid of *B. rigida* × *Dendrocalamus farinosus*

**DOI:** 10.3389/fpls.2023.1231940

**Published:** 2023-09-01

**Authors:** Juan Zhuo, Naresh Vasupalli, Yong Wang, Guoqiang Zhou, Huibin Gao, Ying Zheng, Benxiang Li, Dan Hou, Xinchun Lin

**Affiliations:** ^1^ State Key Laboratory of Subtropical Silviculture, Zhejiang A & F University, Lin’An, Zhejiang, China; ^2^ Forest and Bamboo Resources Conservation and Cultivation Institute, Yibin Forestry and Bamboo Industry Research Institute, Yibin, Sichuan, China; ^3^ Sichuan Changning Century Bamboo Garden, Yibin, Sichuan, China

**Keywords:** natural hybrid bamboo, transcriptomic data, SSR, *GBSSI*, chloroplast genome

## Abstract

Bamboo is one of the fastest-growing plants commonly used in food, fibre, paper, biofuel, ornamental and medicinal industries. Natural hybridization in bamboo is rare due to its long vegetative period followed by gregarious flowering and death of the entire population. In the current study, a new bamboo species, *Bambusa changningensis*, shows intermediate characteristics of *Dendrocalamus farinosus* and *B. rigida* morphologically, but it is unknown whether *B. changningensis* is a natural hybrid. Moreover, *B. changningensis* has been identified as a superior variety of Sichuan Province with high pulping yield, fibre length and width. Therefore, we analyzed the morphological characteristics, DNA markers, DNA barcoding and chloroplast genomes to identify the hybrid origin of *B. changningensis* and possible maternal parent. We have developed the transcriptomic data for *B. changningensis* and mined the SSR loci. The putative parental lines and hybrid were screened for 64 SSR makers and identified that SSR14, SSR28, SSR31 and SSR34 markers showed both alleles of the parental species in *B. changningensis*, proving heterozygosity. Sequencing nuclear gene *GBSSI* partial regions and phylogenetic analysis also confirm the hybrid nature of *B. changningensis*. Further, we have generated the complete chloroplast genome sequence (139505 bp) of *B. changningensis*. By analyzing the cp genomes of both parents and *B. changningensis*, we identified that *B. rigida* might be the female parent. In conclusion, our study identified that *B. changningensis* is a natural hybrid, providing evidence for bamboo’s natural hybridization. This is the first report on confirming a natural bamboo hybrid and its parents through SSR and chloroplast genome sequence.

## 1 Introduction

Bamboos are the only grass and non-timber woody plant species widely distributed across the Americas, Africa, and Asia Pacific (Ramakrishnan et al., 2020). Bamboo plants are vital economic and ecological forestry resources commonly used in papermaking, fuel, food processing, construction and the medicinal industry (Meena et al., 2019; Manandhar et al., 2019). The tribe Bambuseae contains 91 genera and ~1500 bamboo species. According to the number of basic sets of bamboo chromosomes, all bamboo species were divided into four monophyletic lineages with different ploidy levels. The herbaceous bamboos (2n = 20–24) are diploids, neotropical woody bamboos (2n = 40–48), and temperate woody bamboos (2n = 46–48) are tetraploids, whereas the palaeotropical woody bamboos (2n = 70–72) are hexaploids (Zhou et al., 2017a; Guo et al., 2019). Further phylogenomic analyses revealed that woody bamboos originated from complex reticulate evolution, including four extinct diploid lineages over three independent allopolyploid events after the divergence of the herbaceous bamboo lineage (Guo et al., 2019).

Natural hybridization is an important mechanism in plant evolution that periodically stimulates plant speciation (Hegarty and Hiscock, 2005). Combining different genomes in hybrid lineages has broad evolutionary and ecological implications and may promote evolutionary innovation and adaptive expansion (Paun et al., 2009). Bamboo species are monocarpic plants with a prolonged vegetative stage and uncertain flowering cycle (Zheng X. et al., 2020). The flowering cycles of different bamboo species, even those belonging to the same genus, fluctuate considerably. For example, the flowering cycle of *Bambusa* varies from 30 to 150+ years, *Phyllostachys* from 13 to 120 years and *Dendrocalamus* from 8 to 117 years (Zheng X et al., 2020; Hou et al., 2021). Mizuki et al. (2014) estimated the self-pollination rate of three temperate bamboo species at five sites is 96.3%. Therefore, the simultaneous flowering of different bamboo species at the same place and time and the development of hybrid bamboo plants, especially in the natural environment, is rare. Thus, identifying natural bamboo hybrids helps in studying bamboo taxonomy and the origin of bamboo evolution (Goh et al., 2013).

The traditional identification of bamboo species or hybrid plants is mainly based on morphological characteristics such as flowers and bamboo shoots (Lichtenthaler, 1987). Due to the unique flowering characteristics of most bamboo species, it is difficult to rely solely on morphological features, which are unreliable because they are easily influenced by ecological factors (Das et al., 2007). The development of molecular markers and DNA sequencing has brought a new approach to hybrid identification, taxonomy and phylogenetic analysis (Jiang and Zhou, 2014). The DNA marker technology provided a theoretical and practical basis for bamboo breeding and classification and eliminated the errors of traditional classification methods (Zhang and Tang, 2007). Among various kinds of DNA markers, Simple Sequence Repeat (SSR) is the most widely used marker technology in many aspects, such as DNA fingerprinting, genetic map construction, genetic diversity studies, hybrid identification, and genetic resource conservation (Lin et al., 2010; Bhandawat et al., 2016; Cai et al., 2019; Wu et al., 2023). Whereas molecular phylogenetic analysis based on DNA sequencing also provides a powerful method for studying the process and mechanism of hybridization (Horiike, 2016). The nuclear *GBSSI* gene, which encodes granule-bound starch synthase, proved to be more suitable for molecular phylogenetic analysis among bamboo species compared with other nuclear gene fragments (Guo and Li, 2004).

The nuclear genome shows biparental inheritance, whereas the cp genome shows maternal inheritance in angiosperms and patrilineal inheritance in gymnosperms (Wolfe et al., 1987; Pharmawati et al., 2004; Zheng Y et al., 2020). Therefore, the phylogenetic tree of cp DNA often represents a parthenogenetic spectrum. Thus, selecting suitable cytoplasmic molecular markers between the parents and hybrid can determine the maternal and paternal origin in angiosperms and gymnosperms, respectively (Tian and Li, 2002; Zheng et al., 2009). Hence, studying the combination of morphological characters, nuclear and cytoplasmic DNA, has become the criteria for identifying hybrids and their putative parents (Sang and Zhong, 2000).

In the current study, we used three sympodial bamboo species, *Bambusa changningensis, B. rigida* and *D. farinosus*, from China’s Sichuan province. *B. rigida* is one of the native species in Sichuan, considered to have economic and ornamental value with high utilization (Hu et al., 2009). *D. farinosus* is an essential economic sympodial bamboo species in Southwest China. It has essential characteristics such as cold resistance, barren tolerance, and high cellulose content, making it an excellent raw material for pulping and paper making (Jiang et al., 2008). The *B. changningensis* is a newly identified bamboo species that occupies an important position in the bamboo industry in Sichuan province, China with the characteristics of long bamboo shoot period, high bamboo shoot yield, wide adaptability, tolerance to fertilizer and humidity, and also resistance to both abiotic and biotic stress. The phenotypic characteristics of *B. changningensis* are similar or intermediate between the bamboo species *B. rigida* and the *D. farinosus*, considered a potential natural hybrid (Wang et al., 2016). Further, *B. rigida* and the *D. farinosus* were hexaploid bamboo plants containing the same chromosomal number 2n=70 ± 2 (Chen, 2003). Therefore, these plants might be easily crossable, provided they flower at the same time. Moreover, recently, it has been selected for increasing cultivation in Sichuan province because it has superior characteristics like high yield and better pulp quality than other local bamboos (Wang et al., 2020; Zhou et al., 2020).

In 1968, Mr. Daigui Wang, a farmer, identified that *B. rigida* and *D. farinosus* flowered simultaneously at Xinjia, Zhuhai, Changning, Sichuan province, China. He collected the seedlings from the same area and transplanted them near his home the following year. In 2012, Prof. Tongpei identified that along with *B. rigida* and *D. farinosus*, a new bamboo species is also present in those plants raised from seedlings and named it *B. changningensis*, which means similar to *B. rigida* in Chinese (Yi and Li, 2012). Further, he also says that *B. changningensis* might be the hybrid between *B. rigida* and *D. farinosus*, but no evidence exists. Therefore, in the current study, we analyzed the phenotypical characteristics of the *B. changningensis*, *B. rigida* and *D. farinosus*, combined SSR molecular markers, nuclear gene *GBSSI* partial regions and complete chloroplast genomes together to prove the hybrid authenticity of *B. changningensis.*

## 2 Materials and methods

### 2.1 Plant materials and morphological analysis

In this study, we used the young and healthy leaves of *D. farinosus*, *B. changningensis* (R-WTS-BC-005-2015) and *B. rigida* collected from Changning, Sichuan Province, China (28°29’N, 104°58’E). For molecular studies 21 *B. changningensis* individual plant samples collected from three sites were used, whereas three individual plants each for *B. rigida* and *D. farinosus* were used. Further, 15 individuals per species were used for analysing fibre length, fibre width, fibre wall cavity ratio, pulping yield, cellulose content, stem height and stem diameter.

### 2.2 DNA extraction and nuclear sequence amplification

Total genomic DNA was extracted using the CTAB protocol (Doyle, 1991). The total genomic DNA was used as a template for PCR amplification, and the *GBSSI* gene was amplified and sequenced. The primer sequences of *GBSSI* partial regions mentioned by Ye (2010) for PCR amplification (**Table S1**). The PCR amplification was carried out in a 50 μl reaction volume containing 25 μl Taq master mix (2x Specific Taq Master Mix, 250 units, novoprotein, Suzhou, China), 22 μl ddH_2_O, 100 ng total DNA and 2 umol/L of each primer. The PCR program followed was initial denaturation of 95 °C for 5 min, followed by 35 cycles of 95 °C for 30 s (denaturation), 65 °C for 30 s (annealing) and 72 °C for 40 s (extension) and a final extension at 72 °C for 10 min. The PCR products were separated in the 1% agarose gels, and the PCR products were extracted from the gel using SanPrep Column DNA Gel Extraction Kit (Simgen, Hangzhou, China) and sequenced by Sanger sequencing.

### 2.3 Transcriptome sequencing

The total RNA was isolated by Polysaccharides & Polyphenolics-rich Plant Total RNA Kit (Simgen, Hangzhou, China) from the bulk tissues of *B. changningensis* leaves, stem, root and apical meristem as per the manufacturer’s instructions. The quality and quantity of RNA were detected using the NanoPhotometer spectrophotometer (Implen, CA, USA) and electrophoresis (1% agarose gel). The libraries were synthesized utilizing the NEB Next® UltraTM RNA Library Prep Kit for Illumina® (NEB, USA). These constructed libraries were sequenced using the Illumina HiSeq forum to obtain paired-end reads (150 bp). After quality control (QC), transcriptome data were further handled and assembled following the procedure described in (Grabherr et al., 2011) using Trinity v2.8.4. The Assembled results by Trinity were processed using Corset as described in (Davidson and Oshlack, 2014).

### 2.4 Microsatellite analysis

The SSR motifs from the transcriptomic data were mined using the online software MISA (Beier et al., 2017) with 10, 6, 5, 4, 3, and 3 values for mono-, di-, tri-, tetra-, penta- and hexa- SSR motifs, respectively (**Table S2**). The SSR primers were designed by Oligo7 (https://www.oligo.net/downloads.html) (Rychlik, 2007) and synthesized by the Tsingke company (Beijing, China). PCR amplification was performed as described above. The PCR products were separated in the 5% agarose gels, and the PCR products were extracted from the gel using SanPrep Column DNA Gel Extraction Kit (Simgen, Hangzhou, China) and sequenced by Sanger sequencing.

### 2.5 Complete chloroplast genome sequencing and analysis

The total DNA of *B. changningensis* was used to develop the whole genome reads through the Illumina NovaSeq PE150 platform (Novogene Bioinformatics Technology Co. Ltd, Beijing, China). The complete chloroplast genome was assembled by mapping the whole genome reads to reference genome *B. emeiensis* (HQ337797) using MITObim v1.8 (Hahn et al., 2013) and annotated with the PGA (Qu et al., 2019).

### 2.6 Construction of phylogenetic tree

The phylogenetic tree for *GBSSI* partial regions were constructed using PAUP 4.0a software (Swofford, 2008). Twenty-five *GBSSI* sequences from different bamboo species were downloaded from the NCBI database and used in this study (**Table S3a**). The DNA sequences were aligned using MEGAX (Kumar et al., 2018), and highly aligned sequences were used to generate the phylogenetic tree using the maximal parsification method in PAUP 4.0a software as per the parameters described in (Winkworth and Donoghue, 2004).

The 24 cp genomes of different bamboo species were downloaded from the NCBI database (**Table S3b**) and all the genes were extracted and concatenated to get the final sequences matrix to construct a phylogenetic tree. The sequences were aligned by MAFFT (Katoh and Standley, 2013) and the phylogenetic tree was constructed by MEGA X software using the Neighbor-Joining method (Kumar et al., 2018). A bootstrap value of 1000 replicates was used to assess the statistical significance.

## 3 Results

### 3.1 Morphological analysis of putative parents and hybrid plants

**Figure 1:**
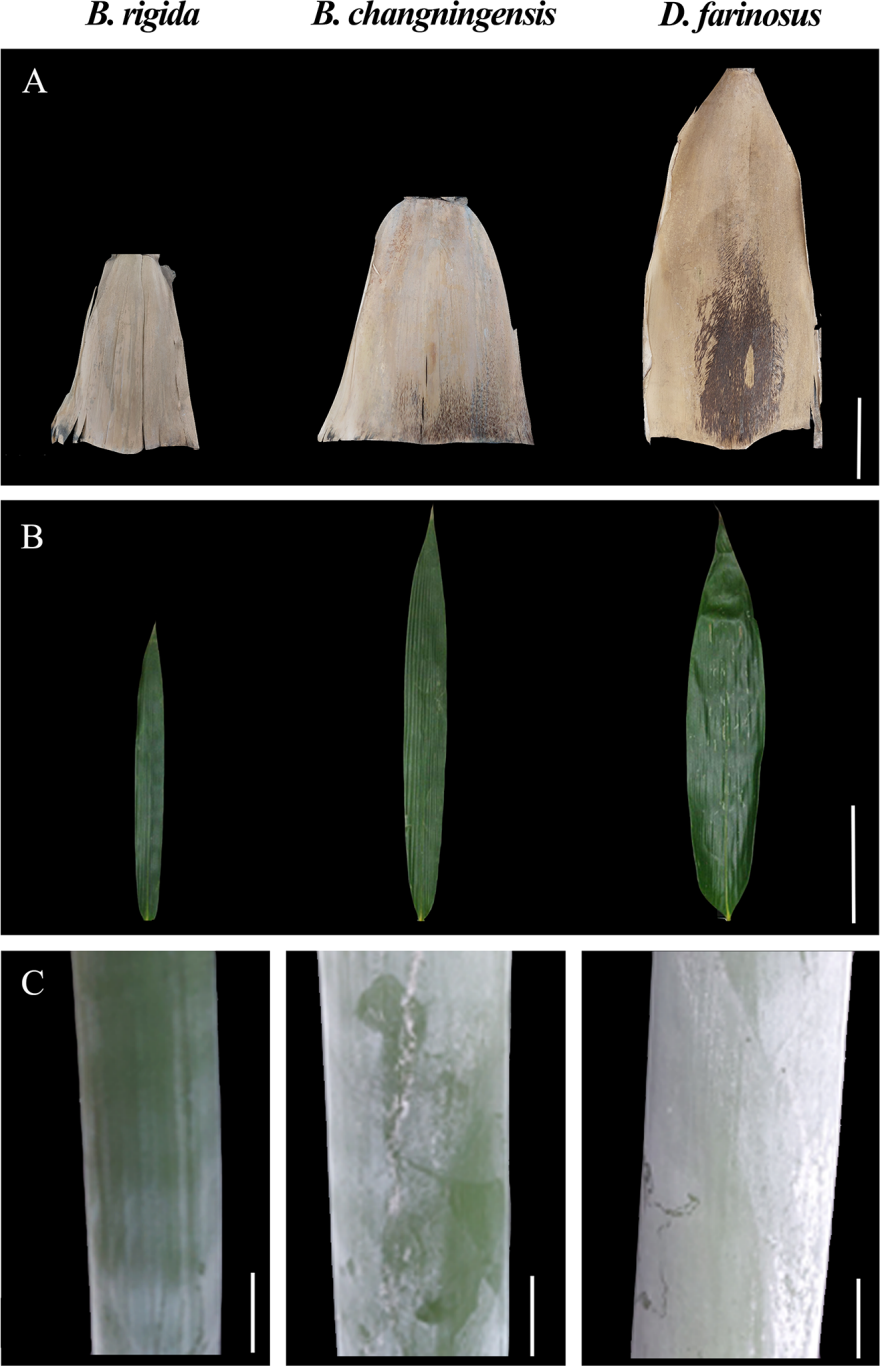
The morphological characteristics comparison of *B. rigida*, *B. changningensis*, and *D. farinosus*. **(A)** bamboo sheath, **(B)** Leaf blade, and **(C)** Stem. Bar size A 50 cm, B 10 cm and C 50 cm.

In this study, we analyzed morphological characteristics such as fibre length, fibre width, fibre wall cavity ratio, pulping yield, cellulose content, stem height, stem diameter, sheath and leaves for *B. changningensis*, *D. farinosus* and *B. rigida* (**Table 1** and **Figure 1**). As expected, *B. changningensis* shows the intermediate characteristics of *D. farinosus* and *B. rigida* in biological traits such as the sheath size, leaf width, and cellulose content. The stem height and diameter were more similar to the putative parent *D. farinosus*. Interestingly, *B. changningensis* also contains superior morphological characteristics like longer fibre length, fibre width, lower fibre wall cavity ratio and higher pulping yield than putative parents *D. farinosus* and *B. rigida*.

**Table 1:** The biological characteristics comparison of *Dendrocalamus farnosus*, *Bambusa changningensis* and *Bambusa rigida*.

Bamboo species	Fiber length (mm)	Fiber width (μm)	Fiber wall cavity ratio (%)	Pulping yield (%)	Stem height (m)	Stem diameter (cm)	Cellulose content (%)
*Dendrocalamus farnosus*	2.35	15.87	3.8	42.6	12-15	6-8	58.84
*Bambusa changningensis*	2.52	22.52	2.28	52.5	10-15	5-8	52.54
*Bambusa rigida*	2.17	18.2	4.61	43.9	5-12	2-6	47.72

### 3.2 Transcriptome analysis and SSR primers screening

**Figure 2:**
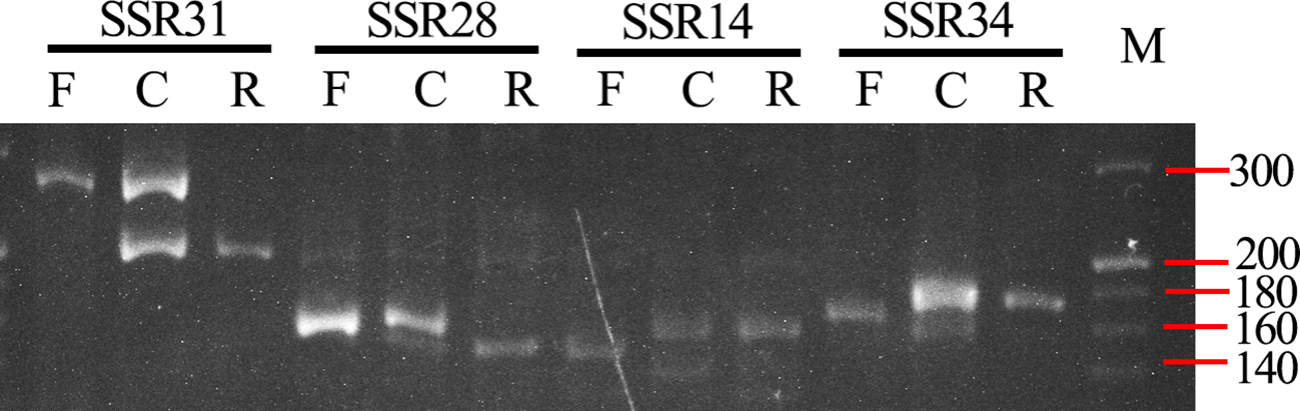
SSR polymorphism between *B. rigida*, *B. changningensis*, and *D. farinosus*. PCR was performed with SSR14, SSR28, SSR31 and SSR34. M. 20 bp ladder.

We have developed pair-end transcriptomic data of *B. changningensis* using the Illumina HiSeq platform to obtain genomic information to develop the DNA markers. A total of 23,270,949 reads containing 6,961,574,574 bp were retained after quality trimming. After *de novo* assembly, a total of 154,983 contigs and 100,364 unigenes were obtained (**Tables S4**, **S5**). The contigs were subjected to SSR mining through MISA software and identified a total of 3,732 SSR loci, including di-, tri-, tetra-, penta- and hexa-nucleotide motifs. The di- and tetra-nucleotide repeats were the most abundant SSR loci detected, accounting for 34.5% and 35.2%, respectively (**Table S2**). Sixty-four sequences containing SSR loci were selected randomly for PCR amplification to identify polymorphism between the *B. changningensis* and its putative parents, *D. farinosus* and *B. rigida* using bulk DNA (**Table S1**). Out of 64 primer pairs, four primer pairs, SSR14, SSR28, SSR31 and SSR34 produced polymorphic PCR amplification (**Figure 2**). The SSR14 primers had an amplification of 139 bp in *D. farinosus*, whereas 144 and ~135 bp bands in *B. rigida*. SSR28 had an amplification size of 168 bp in *D. farinosus*, while *B. rigida* contains 159 and ~180 bp bands. The SSR31 primers had an amplification of 255 bp in *D. farinosus*, whereas 200 bp bands in *B. rigida*. Similarly, The SSR34 primers had an amplification of 160 in *D. farinosus*, whereas 167 bp bands in *B. rigida*. These results confirm that the *B. changningensis* had both putative parental complementary bands, except the ~135 bp bands of *B. rigida*.

**Figure 3:**
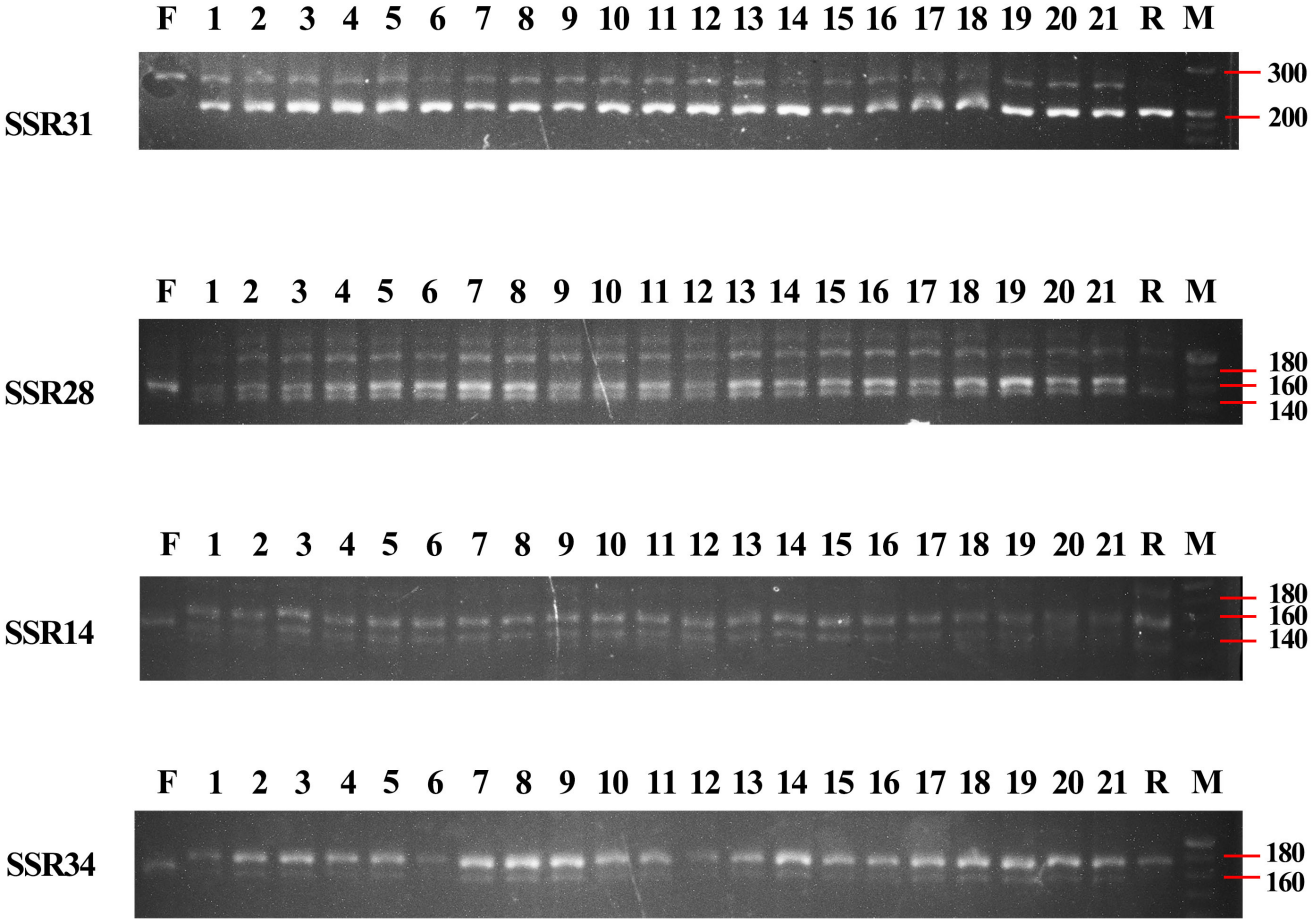
PCR amplification of SSR14, SSR28, SSR31 and SSR34 makers for individuals of *B. changningensis*. F. *D. farinosus*; 1-21. *B. changningensis*; R. *B. rigida*. M. 20 bp DNA ladder.

Moreover, we analysed these four primers in 21 induvial plants of *B. changningensis*, the presence of putative parental bands in all individual plants confirms the heterozygous nature (**Figure 3**). Further, we sequenced the 139, 168, 255, and 160 bp bands in *D. farinosus* and *B. changningensis*, 144, 159, 200, and 167 bp bands in *B. changningensis*, and *B. rigida* to identify the difference in the repeats of the SSR motif. The repeat units of SSR14 were CTCTC and contained a single repeat unit difference between the *D. farinosus* and *B. rigida* (**Figure S1A**). At the same time, SSR28 had three repeat unit differences in the CTC motif (**Figure S1B**). *B. changningensis* contains both putative parental SSR motifs. The repeat unit of SSR31 was AGG and contained a single repeat unit difference between the *D. farinosus* and *B. rigida* (**Figure S1C**). Interestingly, this SSR also has 56 bp single insertion and 1 bp deletion in *D. farinosus*. The repeat units of SSR34 were GCCTC and contained a single repeat unit difference between the *D. farinosus* and *B. rigida* (**Figure S1D**). These results suggest that *B. changningensis* might be the hybrid plant of *D. farinosus* and *B. rigida*.

### 3.3 *GBSSI* gene fragment cloning and evolutionary tree construction

**Figure 4:**
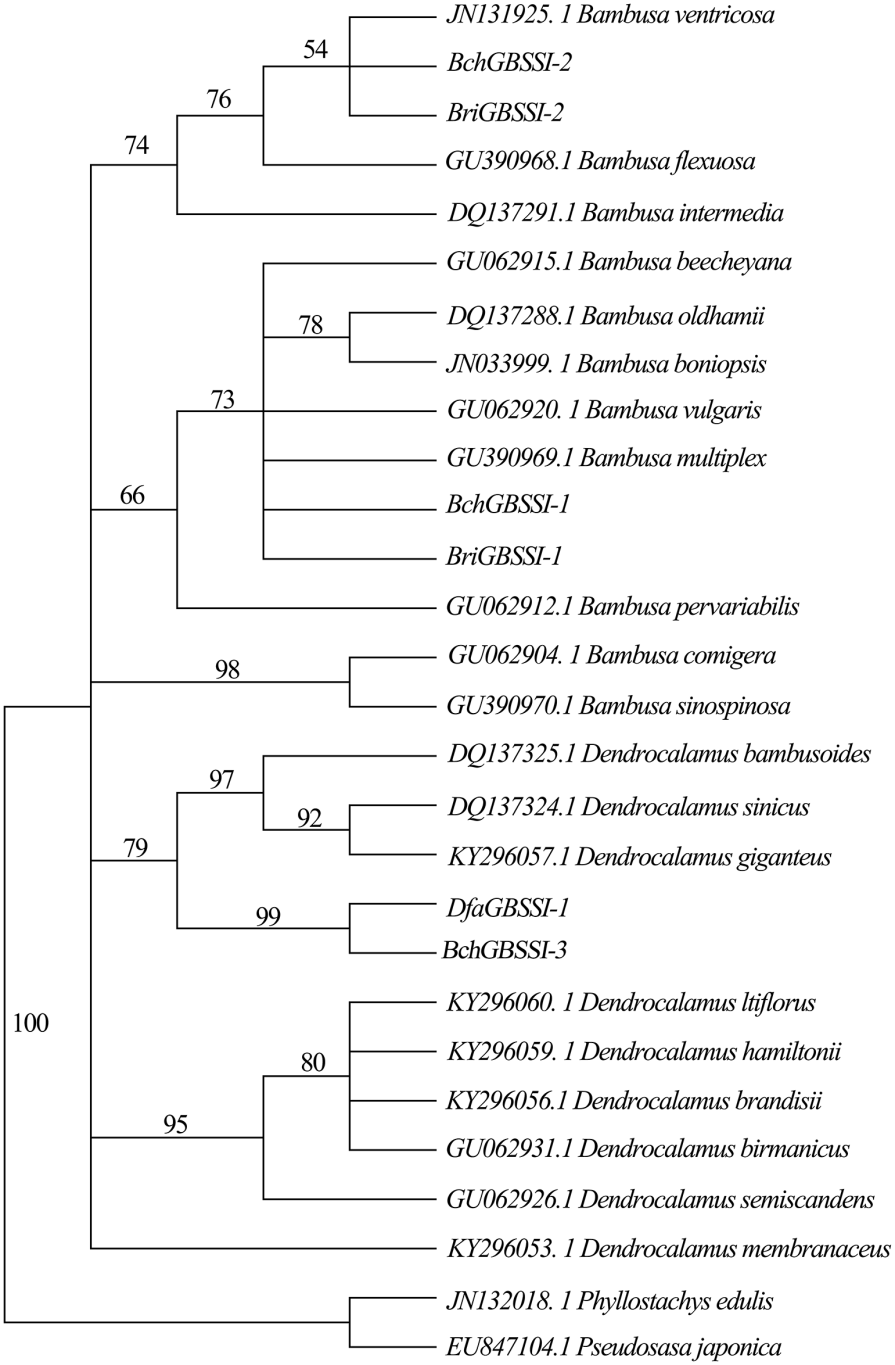
The phylogenetic relationship among *GBSSI* genes of Bambusoideae. The partial DNA sequences of *GBSSI* genes were used to construct the phylogenetic tree. The bootstrap support values are shown on each node. Dfa,. *D. farinosus*; Bch,. *B. changningensis; Bri*, *B. rigida*.

The *GBSSI* gene is about 3 kb in length, and due to the difficulty in PCR amplification, a portion of the sequence (~820 bp) was used for analysis. Using the genomic DNA of *B. changningensis, B. rigida* and *D. farinosus* as a template for amplification, the *GBSSI* gene was amplified. The PCR product was cloned into TA cloning vector PMD18, and 30 clones for each species were sequenced. Sequencing results identified that a total of three different kinds of clones, *BchGBSSI-1*, *BchGBSSI-2* and *BchGBSSI-3*, were present in *B. changningensis*. At the same time, *B. rigida* contains two clones, and *D. farinosus* has only one type of clone, *BriGBSSI-1*, *BriGBSSI-2* and *DfaGBSSI-1*, respectively. Further, we aligned these sequences and identified *BchGBSSI-1* and *BriGBSSI-1*, *BchGBSSI-2* and *BriGBSSI-2*, *BchGBSSI-3* and *DfaGBSSI-1*, which have similar kinds of SNPs. Moreover, a Maximum Parsimonious evolutionary tree was constructed using 23 *GBSSI* sequences downloaded from the NCBI database (**Figure 4**). Similar to the above results, *B. changningensis* two *GBSSI* genes, *BchGBSSI-1* and *BchGBSSI-2*, clustered with *BriGBSSI-1* and *BriGBSSI-2*, respectively, and the 3^rd^ *GBSSI* gene, *BchGBSSI-3* was clustered together with *DfaGBSSI-1*. These results confirm that *B. changningensis* is the hybrid between the parental bamboo species *B. rigida* and *D. farinosus.*

### 3.4 *B. changningensis* complete chloroplast genome sequencing and annotation

**Figure 5:**
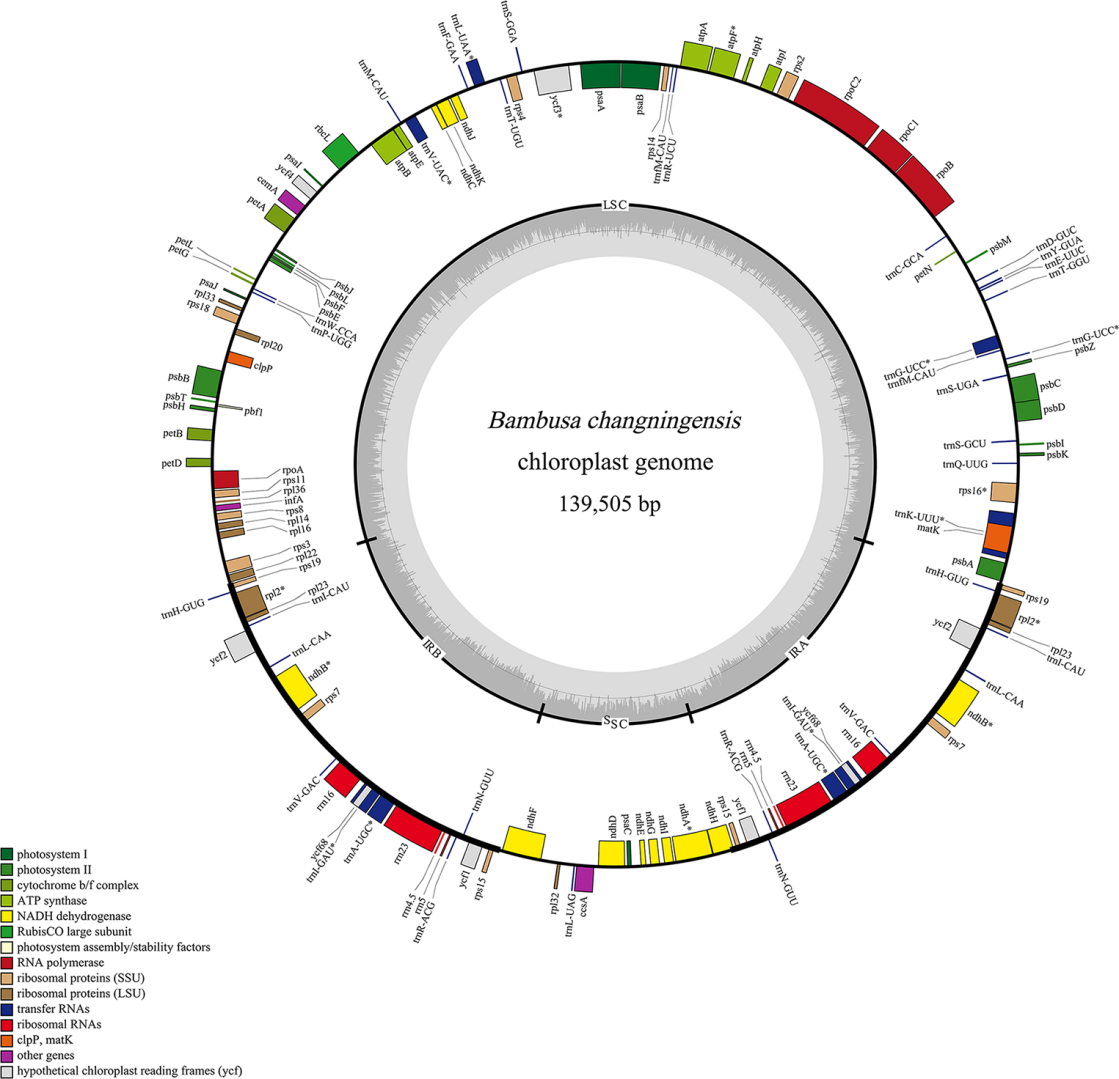
The chloroplast genome of *B. changningensis*. Genes are represented on the inner or outer side of the outer circle to indicate the coding strands.

The complete chloroplast genome of *B. changningensis* was sequenced and analyzed in this study. In the current study, a total of 99,346,872 raw data was developed from the total DNA of *B. changningensis*. After quality trimming, the raw data was reduced to 83416080, and the GC content was 44.98%. Further, the average length of the reads was 150 bp. We mapped the quality reads to the reference cp genome *B. emeiensis* (HQ337797), and 14.69% of the reads were mapped. The complete chloroplast genome sequence of *B. changningensis* (GenBank accession: OM065947) generated was 139505 bp (**Figure 5**). After annotation, a total of 132 genes were found in the cp genome of *B. changningensis*. These include 84 genes coding for essential chloroplast function, 40 tRNA genes, and eight rRNA genes. Further, the cp genome consists of a pair of identical inverted repeat regions of size 21,794 bp separated by a large single-copy (LSC) region of 83,041 bp in size and a small single-copy (SSC) region of 12,876 bp.

### 3.5 Comparison of chloroplast genomes of *B. changningensis*, *D. farinosus* and *B. rigida*

**Figure 6:**
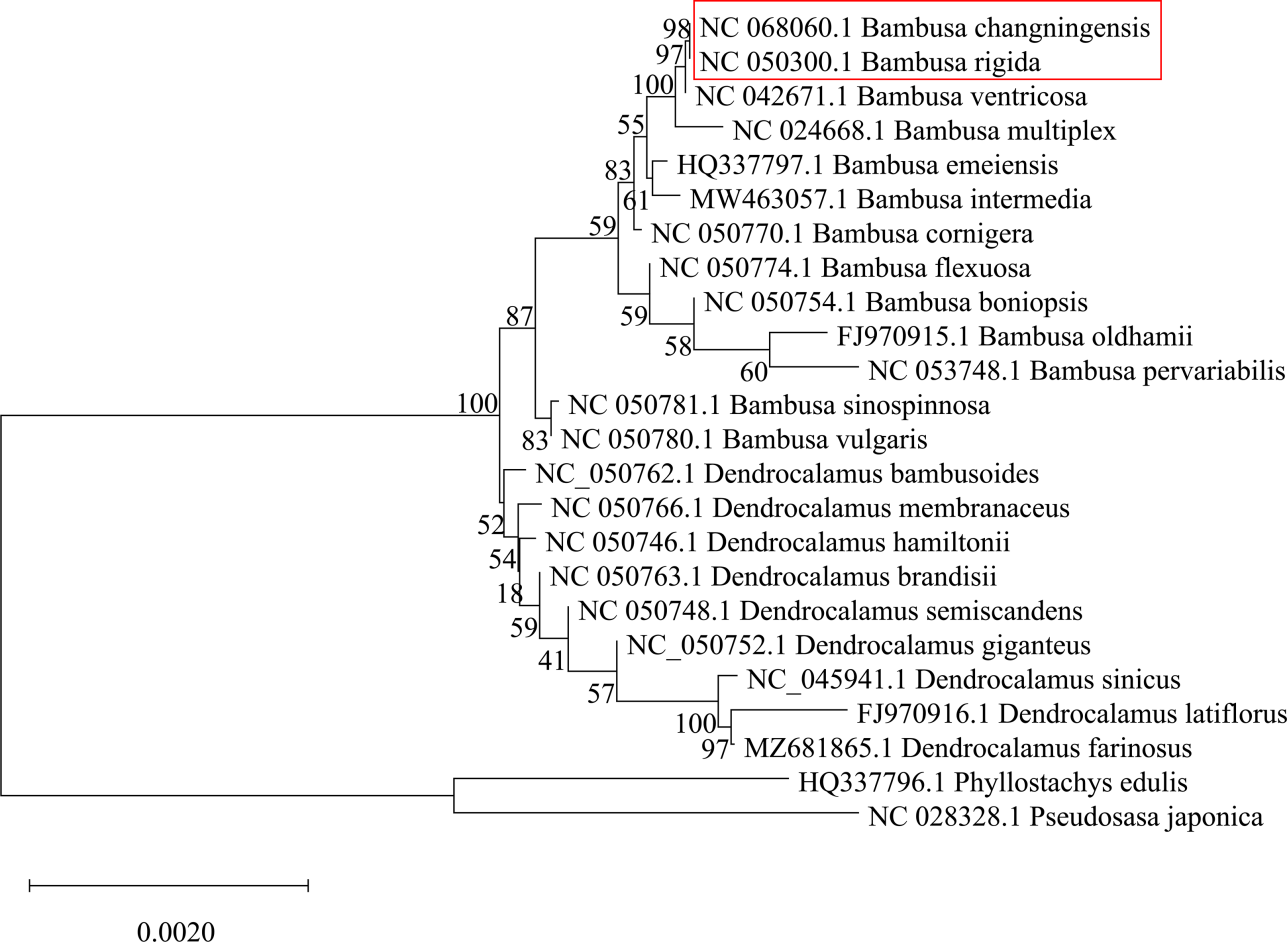
The phylogenetic relationship among extracted gene sequences from complete chloroplast genomes of Bambusoideae. The bootstrap support values are shown on each node.

To identify the female parent of the *B. changningensis*, we aligned the cp genome sequences of *B. changningensis*, *B. rigida* (GenBank accession: MT648824), and *D. farinosus* (GenBank accession: OM177223). The aligned sequences were examined for the presence/absence of Indels/SNPs. Of the 132 cp genes identified in the *B. changningensis*, 33 contain the Indels/SNPs. Among them, 32 genes of the *D. farinosus* displayed Indels/SNPs, whereas the *rpoc2* gene has 8 SNPs and 1 Indel in *D. farinosus*, 4 SNPs and 4 Indels in *B. rigida* and 3 SNPs in *B. changningensis* (**Table 2**). Further, the *D. farinosus* noncoding region displayed 176 SNPs, 47 insertions, and 34 deletions. The largest insertion and deletions are 50 and 31 bp, respectively (**Table S6**). Further, a single bp deletion was also found in *B. rigida*. These results indicate that the cp genomes of *B. changningensis* and *B. rigida* were almost similar, and *D. farinosus* contained SNPs and Indels. Thus *B. rigida* might be the female parent of the hybrid *B. changningensis*. Moreover, we also constructed a phylogenetic tree based on the extracted gene sequences from 24 complete chloroplast genomes using the Neighbor-Joining method with 1,000 bootstrap replicates (**Figure 6**). The hybrid *B. changningensis* was clustered with *B. rigida* with high support values, confirming that *B. rigida* is the female parent of *B. changningensis* and *D. farinosus* is the male parent.

**Table 2:** Details of SNPs and INDELs in the *D. farinosus*, *B. changningensis* and *B. rigida* chloroplast genes.

S.No.	Gene	Dfa	Bch	Bri	SNP	INDEL	S.No.	Gene	Dfa	Bch	Bri	SNP	S.No.	Gene	Dfa	Bch	Bri	SNP
1	*matk*	c	t	t	1		31	*rpoC2*	***ct***	***gc***	***ct***	2	61	*rpoA*	t	c	c	1
2	*matk*	c	t	t	1		32	*rpoC2*	***t***	***c***	***t***	1	62	*rps11*	a	c	c	1
3	*matk*	g	a	a	1		33	*rpoC2*	g	a	a	1	63	*rps8*	t	c	c	1
4	*matk*	g	t	t	1		34	*rpoC2*	a	c	c	1	64	*rps3*	c	t	t	1
5	*matk*	g	t	t	1		35	*rpoC2*	c	a	a	1	65	*rps3*	g	t	t	1
6	*rps16*	c	t	t	1		36	*rpoC2*	g	t	t	1	66	*rps3*	t	g	g	1
7	*rpoB*	a	g	g	1		37	*rpoC2*	g	c	c	1	67	*rps3*	t	c	c	1
8	*rpoB*	a	g	g	1		38	*rps2*	g	a	a	1	68	*rps3*	g	t	t	1
9	*rpoB*	t	c	c	1		39	*atpH*	g	c	c	1	69	*rpl22*	t	c	c	1
10	*rpoB*	t	c	c	1		40	*atpA*	a	g	g	1	70	*rpl22*	c	t	t	1
11	*rpoB*	g	a	a	1		41	*rps14*	a	g	g	1	71	*ndhB*	g	c	c	1
12	*rpoB*	g	a	a	1		42	*psaB*	a	t	t	1	72	*ndhF*	t	a	a	1
13	*rpoB*	t	a	a	1		43	*psaA*	t	c	c	1	73	*ndhF*	a	c	c	1
14	*rpoC1*	c	t	t	1		44	*psaA*	g	a	a	1	74	*ndhF*	a	g	g	1
15	*rpoC1*	c	t	t	1		45	*ndhJ*	a	c	c	1	75	*ndhF*	t	c	c	1
16	*rpoC1*	c	t	t	1		46	*ycf4*	g	c	c	1	76	*ndhF*	c	a	a	1
17	*rpoC2*	a	g	g	1		47	*petA*	g	a	a	1	77	*ndhF*	a	g	g	1
18	*rpoC2*		gat	gat		3	48	*petA*	c	t	t	1	78	*ndhF*	a	g	g	1
19	*rpoC2*	g	a	a	1		49	*petL*	t	a	a	1	79	*ndhF*	g	t	t	1
20	*rpoC2*	g	t	t	1		50	*clpP*	t	c	c	1	80	*rpl32*	c	t	t	1
21	*rpoC2*	g	a	a	1		51	*clpP*	c	t	t	1	81	*ccsA*	g	a	a	1
22	*rpoC2*	**tt**	**tt**			**2**	52	*clpP*	g	t	t	1	82	*ndhD*	c	g	g	1
23	*rpoC2*	**a**	**a**			**1**	53	*psbB*	t	c	c	1	83	*ndhG*	g	a	a	1
24	*rpoC2*	**a**	**a**			**1**	54	*psbB*	a	g	g	1	84	*ndhI*	g	c	c	1
25	*rpoC2*	**a**	**a**			**1**	55	*psbB*	g	a	a	1	85	*ndhA*	t	c	c	1
26	*rpoC2*	***t***	***t***	***c***	*1*		56	*psbB*	a	t	t	1	86	*ndhA*	t	g	g	1
27	*rpoC2*	***a***	***a***	***g***	*1*		57	*psbB*	c	t	t	1	87	*ndhA*	t	a	a	1
28	*rpoC2*	***a***	***a***	***g***	*1*		58	*petB*	c	t	t	1	88	*ndhH*	a	g	g	1
29	*rpoC2*	***a***	***a***	***g***	*1*		59	*petD*	a	g	g	1	89	*ndhB*	c	g	g	1
30	*rpoC2*	***t***	***c***	***t***	1		60	*petD*	c	t	t	1						

*Dfa, *D. farinosus*; Bch, *B. changningensis*; Bri, *B. rigida*; bold brown colour indicates deletion in *B. rigida*, italic bold brown colour indicates SNP in *B. rigida*, and Italic bold black letters indicate SNP in *B. changningensis*. The position of these SNPs and Indels in the chloroplast genome was mentioned in the **Table S6**.

## 4 Discussion

A natural plant hybrid is more common in the natural environment than animals, which is considered a necessary evolutionary process (Rieseberg, 1995; Hegarty and Hiscock, 2005; Zheng et al., 2021). The hybridization is widespread in grass plants (Stebbins, 1956; Singh et al., 2010) but rarely recorded in woody bamboo (Triplett et al., 2010). Because most woody bamboos take a long time to flower, some even as long as 120 years (Janzen, 1976). Therefore, natural bamboo hybridization is considered rare. However, a couple of natural hybridization events are reported in bamboo species based on morphological characteristics suggesting that hybridization events are possible in bamboo, but there is not enough evidence to support this (Maruyama et al., 1979; Muramatsu, 1981). In the current study, we identified a new natural intergeneric bamboo hybrid species *B. changningensis*. Further, we also identified that *B. rigida* is the maternal parent of *B. changningensis*.

### 4.1 Simple sequence repeats results suggest the possibility that *B. changningensis* might be the hybrid of *D. farinosus* and *B. rigida*

SSRs have been widely used in identifying hybrids as a common DNA molecular marker due to the advantages of codominance, simple operation and immunity from environmental effects (Momotaz et al., 2004; Sundaram et al., 2008). After the NGS platform’s discovery, SSR makers’ development became much easier than earlier (Subramanian et al., 2003). In the current study, we have developed the transcriptome data of *B. changningensis*, which can be used for phylogenetic analyses, variety or species identification and molecular plant breeding belonging to the Bambusa genus besides the hybrid identification studies. Earlier, using 15 EST-SSR markers, (Wu et al., 2009) identified four artificial bamboo hybrid species. Further, (Lu et al., 2009) also identified three artificial hybrid bamboos using SSR markers. Similarly, Yuan identified the artificial hybrids derived from *Bambusa multiplex*×*B. chungii* and *B. multiplex* × *Dendrocalamus latiflorus* using SSR markers (Yuan et al., 2021). Likewise, our study also determined that *B. changningensis* is the natural hybrid of *D. farinosus* and *B. rigida*.

### 4.2 Evolutionary tree based on the nuclear gene (*GBSSI* partial regions) provides further evidence for the natural hybrid *Bambusa changningensis*

Molecular phylogenetic studies have shown the surprising ability of gene trees to detect hybrids based on their branching behaviour (Sang and Zhong, 2000; Barraclough and Nee, 2001). *ITS* gene is one of the most widely used genes for phylogenetic analysis at the generic and infrageneric levels in plants (Álvarez and Wendel, 2003). However, some studies have shown that the variation of the *ITS* gene fragment is negligible in woody bamboo, and the taxa cannot be well distinguished (Guo et al., 2001). By contrast, *GBSSI* fragments have more variation than other DNA sequences previously used in woody bamboo (Guo and Li, 2004). Moreover, the *GBSSI* gene exists as a single copy and evolves more quickly in most Gramineae family genomes like rice, wheat, Leymus, Agropyron, and *Arundinarieae* (Soltis et al., 1998; Agafonov et al., 2021). As a single-copy nuclear gene, the *GBSSI* gene has been proven to exhibit higher genetic differentiation in introns of this gene than in *ITS* regions in closely related species (Mason-Gamer et al., 1998; Peralta and Spooner, 2001). Therefore, we chose *GBSSI* partial regions for hybrid identification and phylogenetic studies.

Our study identified three copies of *GBSSI* genes in *B. changningensis*. Among them, two *B. changningensis* genes clustered with two copies of *B. rigida GBSSI* genes, whereas another copy of *B. changningensis GBSSI* gene clustered with *D. farinosus* with strong support. These observations might be due to the hybridization of *B. changningensis* between the parents *B. rigida* and *D. farinosus*. Similar to our results, using the *GBSSI* gene, Goh et al. (2011) also identified a natural bamboo hybrid between *D. pendulus* and *Gigantochloa scortechinii* in Peninsular Malaysia. Further, Spooner identified allopolyploidy in wild potatoes using the *GBSSI* gene (Spooner et al., 2008).

### 4.3 Complete cp genome sequencing and an evolutionary tree based on cp genomes prove that *B. rigida* might be the female parent of *B. changningensis*

Chloroplast DNA is unaffected by genetic recombination, which is of great significance in elucidating the complex phylogenetic relationship of plants (Huang et al., 1994). Further, cp genomes have a maternal inheritance for most angiosperms, and the molecular system reconstruction based on chloroplast genome data is very effective (Gielly and Taberlet, 1994). Therefore, most researchers used cp genes or cp whole genome sequences to identify maternal parents and analyze the genetic relationship. For example, (Nandhini et al., 2013) identified interspecific hybrid *Coffea congensis* × *Coffea canephora* and its parents based on the SNPs present in the *rbc*L and *mat*K. Similarly, (Khew and Chia, 2011) identified the female parent of orchid hybrid *Vanda Miss Joaquim* using the *mat*K. Further, (Uchoi et al., 2016) analyzed the phylogenetic relationship of 23 Citrus genotypes using *rbc*L and *mat*K genes. Chen et al. (2019) used *psbA-trnH*, *matK* and *trnL* sequences as DNA barcoding to establish a rapid identification of *Chrysanthemum indicum* and *Ch. Morifolium*. Yu et al. (2022) identified *Pinus sylvestris* as the paternal parent for *P. funebris* and *P. takahasii* through the complete cp genome (Pinaceae species cp genome inherits paternally). Park et al. (2021) identified the maternal and paternal parents in cumber two inbred lines and their F1 hybrid based on the SNPs and indels present on the cp whole-genome sequences. In our study, to avoid interference between *B. changnengesis* and *B. ventricosa*, we extracted all the genes from 24 cp genomes and concatenate them to get the final sequences matrix to establish the phylogenetic relationship. Our study also identified that *B. rigida* is the maternal parent of the *B. changningensis* based on the SNPs and indels present on the cp whole genome sequence. Interestingly, the *rpoC2* gene contains SNPs or indels in the *B. changningensis* and also in both parents. These results might be because we sequenced the wild species of *B. changningensis* and don’t know the exact individual parents. These minor variations in the SNPs and indels in the cp genome of wild species might be possible (Dally and Second, 2009). The *rpoC2* gene can be considered as the fast-evolving region in these bamboo species that could be used for further evolutionary studies. In conclusion, our results identified that *B. changningensis* is the natural hybrid between the parents *B. rigida* × *D. farinosus*. These findings pave a path toward bamboo speciation and evolutionary studies. The SSR markers developed in this study could be practically used for genotyping of *Bambusa* and *Dendrocalamus* genus plants.

## Data availability statement

The data presented in the study are deposited in the NCBI repository, accession number PRJNA824949 (https://www.ncbi.nlm.nih.gov/genbank/), OM065947 (https://www.ncbi.nlm.nih.gov/genbank/).

## Author contributions

XL, JZ and YZ, DH designed the experiments; JZ performed the experiments; JZ and NV analyzed the data and wrote the manuscript; YW, GZ, HB and BL provided the experimental materials; XL supervised and administrated the project. All the authors have read and approved the final manuscript.

## Supplementary material

Supplementary Figure 1 Alignment of *B. rigida*, *B. changningensis*, and *D. farinosus* allele sequences. **(A)** SSR14; **(B)** SSR28. **(C)** SSR31; **(D)** SSR 34. * Indicates the mutation sites. -Indicates the deletion sites.

## References

[B1] Agafonov A. V. Emtseva M. V. Shabanova E. V. (2021). Microevolutionary relationships between biotypes of *Elymus confusus*, *E. peschkovae*, and *E. sibiricus* (Poaceae) according to hybridization and sequencing of the nuclear gene *GBSS1* (waxy). Bio Web Conf. 38, 6. doi: 10.1051/bioconf/20213800002

[B2] Álvarez I. Wendel J. F. (2003). Ribosomal ITS sequences and plant phylogenetic inference. Mol. Phylogenet. Evol. 29 (3), 417–434. doi: 10.1016/S1055-7903(03)00208-2 14615184

[B3] Barraclough T. G. Nee S. (2001). Phylogenetics and speciation. Trends Ecol. Evol. 16 (7), 391–399. doi: 10.1016/S0169-5347(01)02161-9 11403872

[B4] Beier S. Thiel T. Münch T. Scholz U. Mascher M. (2017). MISA-web: a web server for microsatellite prediction. Bioinformatics. 33 (16), 2583–2585. doi: 10.1093/bioinformatics/btx198 28398459 PMC5870701

[B5] Bhandawat A. Singh G. Raina A. S. Kaur J. Sharma R. K. (2016). Development of genic SSR marker resource from RNA-Seq data in *Dendrocalamus latiflorus* . J. Plant Biochem. Biotechnol. 25 (2), 179–190. doi: 10.1007/s13562-015-0323-9

[B6] Cai K. Zhu L. F. Zhang K. K. Li L. Zhao Z. Y. Zeng W.. (2019). Development and characterization of EST-SSR markers from RNA-Seq data in *Phyllostachys violascens* . Front. Plant Sci. 10, 50. doi: 10.3389/fpls.2019.00050 30774640 PMC6367221

[B7] Chen R. Y. (2003). Chromosome atlas of Chinese principal economic plants IV (Beijing, China: Science Press).

[B8] Chen F. R. Wang T. Guo Q. S. Zhu Z. B. Yang F. Zou Q. J.. (2019). Identification of *Chrysanthemum indicum* in different geographical populations and *Ch. morifolium* based on DNA barcodes of *psbA-trnH*, *matK* and *trnL* . China J. Chin. Materia. Medica. 44 (4), 660–665. doi: 10.19540/j.cnki.cjcmm.2019.0015 30989877

[B9] Dally A. M. Second G. (2009). Chloroplast DNA diversity in wild and cultivated species of rice (Genus *Oryza*, section Oryza). Cladistic-mutation and genetic-distance analysis. Theor. Appl. Genet. 80 (2), 209–222. doi: 10.1007/BF00224389 24220898

[B10] Das M. Bhattacharya S. Basak J. Pal A. (2007). Phylogenetic relationships among the bamboo species as revealed by morphological characters and polymorphism analyses. Biol. Plantarum. 51 (4), 667–672. doi: 10.1007/s10535-007-0140-7

[B11] Davidson N. M. Oshlack A. (2014). Corset: enabling differential gene expression analysis for *de novo* assembled transcriptomes. Genome Biol. 15 (7), 1–14. doi: 10.1186/s13059-014-0410-6 PMC4165373 25063469

[B12] Doyle J. (1991). DNA protocols for plants. Mol. Biol. Evol. 57, 283–293. doi: 10.1007/978-3-642-83962-7_18

[B13] Gielly L. Taberlet P. (1994). The use of chloroplast DNA to resolve plant phylogenies: noncoding versus *rbcL* sequences. Mol. Biol. Evol. 11 (5), 769–777. doi: 10.1016/0303-7207(94)90126-0 7968490

[B14] Goh W. L. Chandran S. Franklin D. C. Isagi Y. Koshy K. C. Sungkaew S.. (2013). Multi-gene region phylogenetic analyses suggest reticulate evolution and a clade of Australian origin among paleotropical woody bamboos (Poaceae: Bambusoideae: Bambuseae). Plant Syst. Evol. 299, 239–257. doi: 10.1007/s00606-012-0718-1

[B15] Goh W. L. Chandran S. Kamiya K. Wong K. M. (2011). A natural hybrid between *Dendrocalamus pendulus* and *Gigantochloa scortechinii* (Poaceae: Bambusoideae: Bambuseae) in Peninsular Malaysia. Gard Bull. Singapore. 62 (2), 223–238. Available at: http://biostor.org/reference/140254.

[B16] Grabherr M. G. Haas B. J. Yassour M. Levin J. Z. Thompson D. A. Amit I.. (2011). Full-length transcriptome assembly from RNA-Seq data without a reference genome. Nat. Biotechnol. 29, 644–652. doi: 10.1038/nbt.1883 21572440 PMC3571712

[B17] Guo Z. H. Chen Y. Y. Li D. Z. Yang J. B. (2001). Genetic variation and evolution of the alpine bamboos (Poaceae: bambusoideae) using DNA sequence data. J. Plant Res. 114 (3), 315–322. doi: 10.1007/PL00013993

[B18] Guo Z. H. Li D. Z. (2004). Phylogenetics of the *Thamnocalamus* group and its allies (Gramineae: Bambusoideae): inference from the sequences of *GBSSI* gene and *ITS* spacer. Mol. Phylogenet. Evol. 30 (1), 1–12. doi: 10.1016/S1055-7903(03)00161-1 15022753

[B19] Guo Z. H. Ma P. F. Yang G. Q. (2019). Genome sequences provide insights into the reticulate origin and unique traits of woody bamboos. Mol. Plant 12 (10), 1353–1365. doi: 10.1016/j.molp.2019.05.009 31145999

[B20] Hahn C. Bachmann L. Chevreux B. (2013). Reconstructing mitochondrial genomes directly from genomic next-generation sequencing reads-a baiting and iterative mapping approach. Nucleic Acids Res. 41 (13), e129. doi: 10.1093/nar/gkt371 23661685 PMC3711436

[B21] Hegarty M. J. Hiscock S. J. (2005). Hybrid speciation in plants: new insights from molecular studies. New Phytol. 165 (2), 411–423. doi: 10.1111/j.1469-8137.2004.01253.x 15720652

[B22] Horiike T. (2016). An introduction to molecular phylogenetic analysis. Rev. Agri Sci. 4, 36–45. doi: 10.7831/ras.4.0_36

[B23] Hou D. Li L. Ma T. F. Pei J. L. Zhao Z. Y. Lu M.. (2021). The *SOC1*-like gene *BoMADS50* is associated with the flowering of *Bambusa oldhamii* . Hortic. Res. 8, 133. doi: 10.1038/s41438-021-00557-4 34059654 PMC8166863

[B24] Hu S. L. Jiang Y. Chen Q. B. Lu X. Q. Sun X. Cao Y.. (2009). Rapd and issr analysis of *bambusa rigida* from the seven different regions in sichuan province. Hubei Agricultural Sciences. doi: 10.14088/j.cnki.issn0439-8114.2009.01.055

[B25] Huang Y. Li C. L. Ma C. Wu N. H. (1994). Chloroplast DNA and its application to plant systematic studies. Chin. Bull. Bot. 11 (2), 11–25. doi: 10.1007/bf02344252

[B26] Janzen D. H. (1976). Why bamboos wait so long to flower. Annu. Rev. Ecol. Syst. 7, 347–391. doi: 10.1146/annurev.es.07.110176.002023

[B27] Jiang Y. Hu S. L. Cheng Q. B. Lu X. Q. Li Y. Cao Y.. (2008). Studies on genetic diversity of *Dendrocalamus farinosus* from the different regions in Sichuan Province by RAPD and ISSR markers. J. Fujian Coll. Forestry. 28 (3), 276–280. doi: 10.1016/S1872-2040(08)60061-4

[B28] Jiang K. Y. Zhou M. B. (2014). Recent advances in bamboo molecular biology. J. Trop. Subtrop. Bot. 22 (6), 632–642. doi: 10.11926/j.issn.1005-3395.2014.06.012

[B29] Katoh K. Standley D. M. (2013). MAFFT multiple sequence alignment software version 7: improvements in performance and usability. Mol. Biol. Evol. 30 (4), 772–780. doi: 10.1093/molbev/mst010 23329690 PMC3603318

[B30] Khew G. S. W. Chia T. F. (2011). Parentage determination of Vanda Miss Joaquim (Orchidaceae) through two chloroplast genes *rbcL* and *matK* . AoB Plants, plr018. doi: 10.1093/aobpla/plr018 22476488 PMC3156982

[B31] Kumar S. Stecher G. Li M. Knyaz C. Tamura K. (2018). MEGA X: molecular evolutionary genetics analysis across computing platforms. Mol. Biol. Evol. 35 (6), 1547–1549. doi: 10.1093/molbev/msy096 29722887 PMC5967553

[B32] Lichtenthaler H. K. (1987). Chlorophylls and carotenoids: Pigments of photosynthetic biomembranes. Meth Enzymol. 148, 350–382. doi: 10.1016/0076-6879(87)48036-1

[B33] Lin X. C. Lou Y. F. Liu J. Peng J. S. Liao G. L. Fang W. (2010). Crossbreeding of Phyllostachys species (Poaceae) and identification of their hybrids using ISSR markers. GMR. 9 (3), 1398–1404. doi: 10.4238/vol9-3gmr855 20662154

[B34] Lu J. J. Katsuhiko Y. Fang W. Tang D. Q. (2009). Identification of the hybrid bamboo F1 by SSR markers. Scientia Silvae Sinicae. 45 (3), 29–34. doi: 10.1007/978-1-4020-9623-5_5

[B35] Manandhar R. Kim J. H. Kim J. T. (2019). Environmental, social and economic sustainability of bamboo and bamboo-based construction materials in buildings. J. Asian Archit. Build. 18 (2), 49–59. doi: 10.1080/13467581.2019.1595629

[B36] Maruyama I. Okamura H. Murata G. (1979). On a new hybrid genus Hibanobambusa. Acta Phytotax. Geobot. 30, 148–152. doi: 10.18942/bunruichiri.KJ00001078327

[B37] Mason-Gamer R. J. Weil C. F. Kellogg E. A. (1998). Granule-bound starch synthase: structure, function, and phylogenetic utility. Mol. Biol. Evol. 15 (12), 1658–1673. doi: 10.1093/oxfordjournals.molbev.a025893 9866201

[B38] Meena R. K. Bhandhari M. S. Barhwal S. Ginwal H. S. (2019). Genetic diversity and structure of *Dendrocalamus hamiltonii* natural metapopulation: a commercially important bamboo species of northeast Himalayas. 3 Biotech. 9, 60. doi: 10.1007/s13205-019-1591-1 PMC6356152 30729084

[B39] Mizuki I. Sato A. Matsuo A. Suyama Y. Suzuki J. I. Makita A. (2014). Clonal structure, seed set, and self-pollination rate in mass-flowering bamboo species during off-year flowering events. PloS One 9 (8), e105051. doi: 10.1371/journal.pone.0105051 25115953 PMC4130643

[B40] Momotaz A. Forster J. W. Yamada T. (2004). Identification of cultivars and accessions of Lolium, Festuca and Festulolium hybrids through the detection of simple sequence repeat polymorphism. Plant Breeding. 123 (4), 370–376. doi: 10.1111/j.1439-0523.2004.00962.x

[B41] Muramatsu M. (1981). Hybridization among Bambusaceae species. In Bamboo production and utilization, 65–69. Proceedings of the Congress Group 5.3A, Production and utilization of bamboo and related species, XVII IUFRO [International Union of Forestry Research Organization] World Congress, Kyoto, Japan. IUFRO, Vienna, Austria.

[B42] Nandhini R. B. Rahul R. N. Thilaga S. Rao N. S. P. Ganesh D. (2013). Molecular distinction of C× R hybrid (*Coffea congensis*× *Coffea canephora*) from morphologically resembling male parent using *rbcL* and *matK* gene sequences. S Afr J. Bot. 88, 334–340. doi: 10.1016/j.sajb.2013.08.011

[B43] Park H. S. Lee W. K. Lee S. C. Lee H. O. Joh H. J. Park J. Y.. (2021). Inheritance of chloroplast and mitochondrial genomes in cucumber revealed by four reciprocal F1 hybrid combinations. Sci. Rep. 11 (1), 2506. doi: 10.1038/s41598-021-81988-w 33510273 PMC7843999

[B44] Paun O. Forest F. Fay M. F. Chase M. K. (2009). Hybrid speciation in angiosperms: parental divergence drives ploidy. New Phytol. 182, 507–518. doi: 10.1111/j.1469-8137.2009.02767.x 19220761 PMC2988484

[B45] Peralta I. E. Spooner D. M. (2001). Granule-bound starch synthase (*GBSSI*) gene phylogeny of wild tomatoes (*Solanum L. section Lycopersicon* [Mill.] Wettst. subsection *Lycopersicon*). Am. J. Bot. 88 (10), 1888–1902. doi: 10.2307/3558365 21669622

[B46] Pharmawati M. Yan G. Sedgley R. Finnegan P. M. (2004). Chloroplast DNA inheritance and variation in *Leucadendron* species (Proteaceae) as revealed by PCR-RFLP. Theor. Appl. Genet. 109, 1694–1701. doi: 10.1007/s00122-004-1800-z 15365629

[B47] Qu X. J. Moore M. J. Li D. Z. Yi T. S. (2019). PGA: a software package for rapid, accurate, and flexible batch annotation of plastomes. Plant Methods 15, 50. doi: 10.1186/s13007-019-0435-7 31139240 PMC6528300

[B48] Ramakrishnan M. Yrjälä K. Vinod K. K. Sharma A. Cho J. Satheesh V.. (2020). Genetics and genomics of moso bamboo (*Phyllostachys edulis*): Current status, future challenges, and biotechnological opportunities toward a sustainable bamboo industry. Food Energy Secur. 9 (4), e229. doi: 10.1002/fes3.229

[B49] Rieseberg L. H. (1995). The role of hybridization in evolution: old wine in new skins. Am. J. Bot. 82 (7), 944–953. doi: 10.1002/j.1537-2197.1995.tb15711.x

[B50] Rychlik W. (2007). OLIGO 7 primer analysis software. Methods Mol. Biol. 402, 35–39. doi: 10.1007/978-1-59745-528-2_2 17951789

[B51] Sang T. Zhong Y. (2000). Testing hybridization hypotheses based on incongruent gene trees. Syst. Biol. 49 (3), 422–434. doi: 10.1080/10635159950127321 12116420

[B52] Singh A. K. Singh L. Kumar C. Kumar P. Dimree S. K. (2010). Para grass hybrid (*Brachiaria* sp.) – A potential forage for India. Environ. Ecol. 28 (3), 1602–1606. doi: 10.1109/78.875477

[B53] Soltis D. E. Soltis P. S. Doyle J. J. (1998). Molecular Systematics of Plants II: DNA Sequencing. London: Chapman and Hall Press. doi: 10.1007/978-1-4615-5419-6

[B54] Spooner D. M. Rodríguez F. Polgár Z. Ballard H. E. Jansky S. H. (2008). Genomic origins of potato polyploids: *GBSSI* gene sequencing data. Crop Science. 48 (S1), S27–S36. doi: 10.2135/cropsci2007.09.0504tpg

[B55] Stebbins G. L. (1956). Cytogenetics and evolution of the grass family. Am. J. Bot. 43 (10), 890–905. doi: 10.1002/j.1537-2197.1956.tb11182.x

[B56] Subramanian S. Mishra R. K. Singh L. (2003). Genome-wide analysis of microsatellite repeats in humans: their abundance and density in specific genomic regions. Genome Biol. 4 (2), 1–10. doi: 10.1186/gb-2003-4-2-r13 PMC151303 12620123

[B57] Sundaram R. M. Naveenkumar B. Biradar S. K. Balachandran S. M. Mishra B. IlyasAhmed M.. (2008). Identification of informative SSR markers capable of distinguishing hybrid rice parental lines and their utilization in seed purity assessment. Euphytica. 163, 215–224. doi: 10.1007/s10681-007-9630-0

[B58] Swofford D. L. (2008). PAUP (phylogenetic analysis using parsimony). In: Encyclopedia of Genetics, Genomics, Proteomics and Informatics. Dordrecht: Springer. doi: 10.1007/978-1-4020-6754-9_12413

[B59] Tian X. Li D. Z. (2002). Application of DNA sequences in plant phylogenetic study. Acta Botanica Yunnanica. 24 (2), 170–184. doi: 10.3969/j.issn.2095-0845.2002.02.004

[B60] Triplett J. K. Oltrogge K. A. Clark L. G. (2010). Phylogenetic relationships and natural hybridization among the North American woody bamboos (Poaceae: Bambusoideae: *Arundinaria*). Am. J. Bot. 97 (3), 471–492. doi: 10.3732/ajb.0900244 21622410

[B61] Uchoi A. Malik S. K. Choudhary R. Kumar S. Rohini M. R. Pal D.. (2016). RETRACTION: Inferring Phylogenetic Relationships of Indian Citron (*Citrus medica* L.) based on *rbcL* and *matK* Sequences of Chloroplast DNA. Biochem. Genet. 54 (3), 249–269. doi: 10.1007/s10528-019-09939-9 26956119

[B62] Wang Y. Lei Y. C. Chen P. Zhou G. Q. Yu Y. (2020). Analysis of Fiber Morphology and Pulping Properties of *Bambusa changningensis* Yi et B. X. Li. Word Bamboo And Rattan. 18 (5), 51.

[B63] Wang Y. Zhou G. Q. Jia T. B. He X. B. Hu Y. B. (2016). Studies on biological and ecological characteristics of *Bambusa Changningsis* . J. Sichuan For Sci. Tech. 37 (06), 94–96. doi: 10.16779/j.cnki.1003-5508.2016.06.021

[B64] Winkworth R. C. Donoghue M. J. (2004). Viburnum phylogeny: evidence from the duplicated nuclear gene *GBSSI* . Mol. Phylogenet. Evol. 33 (1), 109–126. doi: 10.1016/j.ympev.2004.05.006 15324842

[B65] Wolfe K. H. Li W. H. Sharp P. M. (1987). Rates of nucleotide substitution vary greatly among plant mitochondrial, chloroplast, and nuclear DNAs. PNAS. 84 (24), 9054–9058. doi: 10.1073/pnas.84.24.9054 3480529 PMC299690

[B66] Wu M. D. Dong W. J. Tang D. Q. (2009). Identification of four caespitose hybrid bamboos by using SSR markers. Mol. Plant Breeding. 7 (5), 959–965. doi: 10.3969/mpb.007.000959

[B67] Wu Y. D. Wang Y. Fan X. C. Zhang Y Jiang J. F. Sun L. (2023). QTL mapping for berry shape based on a high-density genetic map constructed by whole-genome resequencing in grape. Hortic. Res. 9 (4), 729–742. doi: 10.1016/J.HPJ.2022.11.005

[B68] Ye J. (2010). A preliminary study on the origin of natural hybridization of *Bambusa subgen* . Dendrocalamopsis. [master’s thesis] [China(Beijing)]: Chinese Academy of Sciences.

[B69] Yi T. P. Li B. X. (2012). *Bambusa Changningensis* Yi et B.X.Li——a new Species (Babusoideae, Poaceae) of Economical Bamboos in China. J. Sichuan For. Sci. Technology. 33 (3), 7–10. doi: 10.3969/j.issn.1003-5508.2012.03.002

[B70] Yu T. Jia Z. Y. Dayananda B. Li J. Q. Guo X. L. Shi L.. (2022). Analysis of the chloroplast genomes of four Pinus species in Northeast China: Insights into hybrid speciation and identification of DNA molecular markers. J. For. Res. 33, 1881–1890. doi: 10.1007/s11676-021-01432-7

[B71] Yuan J. Ma J. X. Zhong Y. B. Yue J. J. (2021). SSR-based hybrid identification, genetic analyses and fingerprint development of hybridization progenies from sympodial bamboo (Bambusoideae, Poaceae). J. Nanjing For Univ(Nat Sci. Ed). 45 (5), 10–18. doi: 10.12302/j.issn.1000-2006.202012046

[B72] Zhang S. Tang D. Q. (2007). A review on application of DNA marker in bamboos and its limits. J. Bamboo Res. 26 (1), 10–14. doi: 10.3969/j.issn.1000-6567.2007.01.003

[B73] Zheng W. Gao D. H. Wang L. Y. (2009). Review of plant hybrid origination and its research progress. J. Anhui Agri Sci. 37 (22), 10378–10381. doi: 10.3969/j.issn.0517-6611.2009.22.022

[B74] Zheng Y. Hou D. Zhuo J. Zheng R. H. Wang Y. Li B. X.. (2020). Complete chloroplast genome sequence of *Bambusa rigida* (Bambuseae). Mitochondrial DNA B Resour 5 (3), 2972–2973. doi: 10.1080/23802359.2020.1793699 33458020 PMC7781895

[B75] Zheng X. Lin S. Y. Fu H. J. Wan Y. W. Ding Y. L. (2020). The bamboo flowering cycle sheds light on flowering diversity. Front. Plant Sci. 11, 381. doi: 10.3389/fpls.2020.00381 32362903 PMC7180196

[B76] Zheng W. Yan L. J. Burgess K. S. Luo Y. H. Zou J. Y. Qin H. T.. (2021). Natural hybridization among three Rhododendron species (Ericaceae) revealed by morphological and genomic evidence. BMC Plant Biol. 21 (1), 1–12. doi: 10.1186/s12870-021-03312-y 34763662 PMC8582147

[B77] Zhou G. Q. Wang Y. Jia T. B. (2020). Superior Clone Selection and Breeding for *Bambusa changningensis* Yi et B. X. Li. Word Bamboo And Rattan. 18 (4), 30–34. doi: 10.12168/sjzttx.2020.04.006

[B78] Zhou M. Xu C. Shen L. Xiang W. Tang D. (2017a). Evolution of genome sizes in Chinese Bambusoideae (Poaceae) in relation to karyotype. Trees. 31, 41–48. doi: 10.1007/s00468-016-1453-y

