# Supplementary figures and images for: Molecular identification of Bambusa changningensis is the natural bamboo hybrid of B. rigida × Dendrocalamus farinosus
Source: Front Plant Sci. 2023 Sep 1;14:1231940. doi: 10.3389/fpls.2023.1231940 (PMC10505617; doi:10.3389/fpls.2023.1231940)

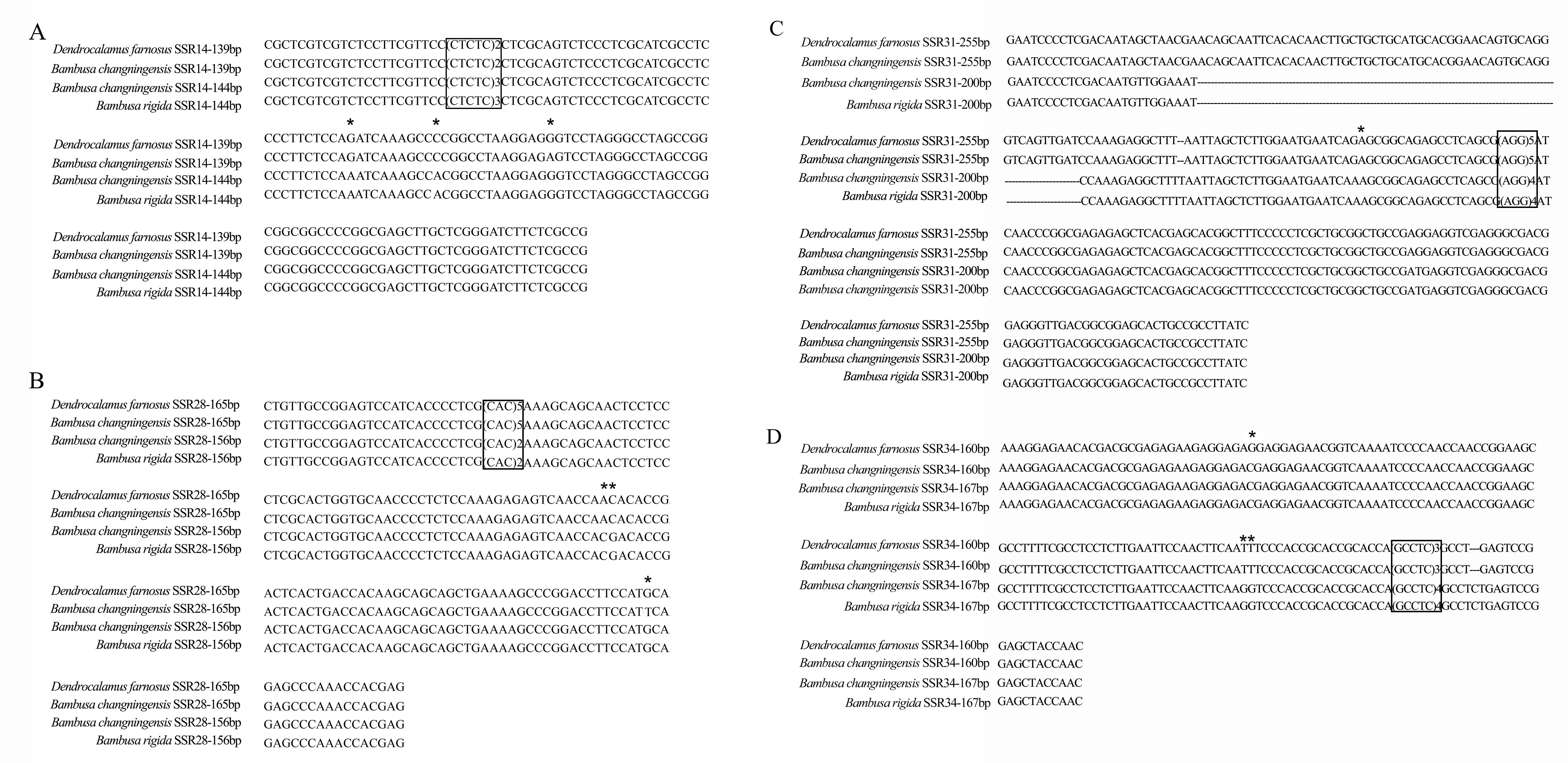

Supplement: Supplementary Figure 1 — Alignment of B. rigida, B. changningensis, and D. farinosus allele sequences. (A) SSR14; (B) SSR28. (C) SSR31; (D) SSR 34. * Indicates the mutation sites. -Indicates the deletion sites. [file Image_1.tif]
