# Supplementary material for: Molecular identification of Bambusa changningensis is the natural bamboo hybrid of B. rigida × Dendrocalamus farinosus
Source: Front Plant Sci. 2023 Sep 1;14:1231940. doi: 10.3389/fpls.2023.1231940 (PMC10505617; doi:10.3389/fpls.2023.1231940)
Supplement: Supplementary file 5 [file DataSheet_4.docx]

**Table S4. Length distribution of contigs and unigenes**

| **Nucleotides length (bp)** | **Contigs** | **Unigenes** |
| --- | --- | --- |
| 200-300 | 40,129 | 37,712 |
| 301-500 | 35,118 | 27,342 |
| 501-1000 | 36,275 | 18,666 |
| 1001-2000 | 31,209 | 11,357 |
| >2000 | 12,252 | 5,287 |
| Total Number | 154,983 | 100,364 |
| Total Length (bp) | 127,591,434 | 63,910,774 |
| N50 length (bp) | 957 | 1,288 |
| Mean length (bp) | 637 | 823 |
